# S-Thiolation Targets Albumin in Heart Failure

**DOI:** 10.3390/antiox9080763

**Published:** 2020-08-17

**Authors:** Maura Brioschi, Erica Gianazza, Alice Mallia, Beatrice Zoanni, Alessandra Altomare, Alma Martinez Fernandez, Piergiuseppe Agostoni, Giancarlo Aldini, Cristina Banfi

**Affiliations:** 1 Centro Cardiologico Monzino, IRCCS, 20138 Milano, Italy; maura.brioschi@ccfm.it (M.B.); erica.gianazza@ccfm.it (E.G.); alice.mallia@ccfm.it (A.M.); beatrice.zoanni@ccfm.it (B.Z.); alma.martinez@ccfm.it (A.M.F.); piergiuseppe.agostoni@ccfm.it (P.A.); 2 Dipartimento di Scienze Farmaceutiche, Università di Milano, 20133 Milano, Italy; alessandra.altomare@unimi.it (A.A.); giancarlo.aldini@unimi.it (G.A.); 3 Dipartimento di Scienze Cliniche e di Comunità, Sezione Cardiovascolare, Università di Milano, 20122 Milano, Italy

**Keywords:** s-thiolation, albumin, heart failure, oxidative stress

## Abstract

Human serum albumin (HSA) is associated with several physiological functions, such as maintaining oncotic pressure and microvascular integrity, among others. It also represents the major and predominant antioxidant in plasma due to the presence of the Cys34 sulfhydryl group. In this study, we assessed qualitative and quantitative changes in HSA in patients with heart failure (HF) and their relationship with the severity of the disease. We detected by means of mass spectrometry a global decrease of the HSA content in the plasma of HF patients in respect to control subjects, a significant increase of thio-HSA with a concomitant decrease in the reduced form of albumin. Cysteine and, at a lesser extent, homocysteine represent the most abundant thiol bound to HSA. A strong inverse correlation was also observed between cysteine-HSA and peak VO_2_/kg, an index of oxygen consumption associated with HF severity. Moreover, in HL-1 cardiomyocytes incubated with H_2_O_2_, we showed a significant decrease of cell viability in cells treated with thio-HSA in respect to restored native-HSA. In conclusion, we found for the first time that S-thiolation of albumin is increased in the plasma of HF patients and induced changes in the structure and antioxidant function of HSA, likely contributing to HF progression.

## 1. Introduction

Human serum albumin (HSA), the most abundant circulatory protein, is associated with several vital physiological functions, such as maintaining oncotic pressure and microvascular integrity, and regulating metabolic and vascular functions [1]. Further, albumin transports metals, fatty acids, cholesterol, bile pigments, and drugs. It also represents the major and predominant antioxidant in plasma [2], a body compartment exposed to continuous oxidative stress where only a limited amount of antioxidant enzymes are available [3]. Albumin exerts its antioxidant activity by acting through its multiple-binding sites and free radical-trapping properties. For example, by binding to HSA, copper and iron are less susceptible to participating in the Fenton reaction, which leads to the formation of the deleterious hydroxyl radical [2]. Although, in plasma, most of the copper is bound to ceruloplasmin, a high percentage of the metal ion can bind to albumin due to the presence of the high-affinity site for copper, the N-terminal tripeptide Asp-Ala-His [4]. As far as concerns the free-radical-trapping properties, HSA exerts its antioxidant activity mainly through the presence of the reduced Cys34. [3]. The Cys34 sulfhydryl group present on albumin represents the largest fraction (~80%, corresponding to ~500 µmol/L) of all free thiols in plasma, thus conferring to HSA, due to its abundance, a major role in the plasma antioxidant capacity [2]. In plasma of healthy young subjects, 70–80% of total HSA presents Cys34 as a free sulfhydryl group, whereas the 20–30% of the Cys34 forms a reversible mixed disulphide with low-molecular-weight thiols, like cysteine, homocysteine, glutathione, or cysteinylglycine, leading to the formation of S-thiolated albumin [5,6]. Acting as a free radical scavenger, the Cys34 residue traps multiple reactive oxygen species (ROS) and reactive nitrogen species (RNS) [2,6]; it is oxidized by the two-electron oxidants, hydrogen peroxide and peroxynitrite, and is also the target for one-electron oxidants, such as the hydroxyl radical, the carbonate anion radical, and nitrogen dioxide [7]. The evidence that albumin oxidation occurs in vivo has been reported only recently thanks to the advent of mass spectrometry (MS) techniques, together with chromatographic analytical approaches, which allowed the detection of oxidative modifications of albumin. Because these oxidized forms of albumin are not present when the protein is secreted from hepatocytes, they have been thus proposed as potential biomarkers of the involvement of the oxidative stress/damage in human disease and the antioxidant activity of the albumin Cys34. Many studies have contributed to highlighting that the ratio of reduced/oxidized form of HSA is related to age [8] and pathological conditions (reviewed in [3]). Moreover, the quality and integrity of HSA molecules in dialysis patients are altered, with an impairment of the free radical-scavenging ability and reduced drug-binding properties [9,10]. Further, the levels of oxidized albumin are a positive predictor of cardiovascular mortality in normoalbuminemic hemodialysis patients, especially in those with pre-existing cardiovascular diseases (CVDs) [11]. On the other hand, in patients with acute lung injury, albumin administration favorably influenced plasma thiol-dependent antioxidant status and decreased levels of protein oxidative damage [12]. Finally, an increased risk in all-cause mortality and cardiovascular mortality is usually associated with a low serum albumin concentration [13,14]. All the above-mentioned biological functions of albumin, including the antioxidant capacity, the maintenance of vascular integrity and colloid osmotic pressure, and the binding and transport of endogenous and exogenous substances [15], could be all implicated in the pathogenesis of CVDs. Hypoalbuminemia is prevalent in patients with heart failure (HF), and it appears to be reliable in predicting mortality among individuals with HF and a reduced ejection fraction (<40%), both in acute and chronic states [16,17]. Finally, in elderly patients without HF, hypoalbuminemia was found to be significantly associated with incident HF [18,19]. In this study, we assessed qualitative and quantitative changes in serum albumin in HF patients, which may ultimately contribute to the development or progression of the disease. 

## 2. Materials and Methods

### 2.1. Study Population

Plasma samples were obtained from a subset of healthy subjects (controls) and HF patients matched according to their age, sex, and clinical characteristics. The study was approved by the Ethical Committee European Institute of Oncology and Monzino Cardiologic Center (registration number R205-CCFM S208/412 and R454/16-CCM470) and all patients gave their informed consent before taking part in the study. All patients belong to a cohort of HF patients regularly followed at our HF unit and underwent our standard HF assessment, which included full clinical evaluation, standard laboratory tests, echocardiography, spirometry, and alveolar-capillary diffusion, as well as a cardiopulmonary exercise test, as previously described [20]. All patients were in stable clinical conditions. 

### 2.2. Quantitation of S-Thiolated Albumin by Mass Spectrometry (MS)

The relative composition of albumin isoforms in human plasma samples was evaluated, as previously described [21,22], by direct infusion using the Xevo TQ-S micro triple quadrupole mass spectrometer coupled with the ACQUITY UPLC^®^ M-Class system (Waters Corporation, Milford, CT, USA). Briefly, centrifuged plasma samples at 3000× *g* for 10 min at 4 °C were diluted 200-fold in 50% ACN containing 0.1% formic acid (FA). After centrifugation at 14,000× *g* for 10 min at 4 °C, 5 µL were injected in full loop mode at 5 µL/min and the spectra were acquired for 6 min with the following parameters: positive ESI mode; mass range 1100–1350 *m*/*z*; MS scan in multi-channel acquisition (MCA) with 1 s scan time; capillary voltage 3 kV; cone 90 V; desolvation temperature 350 °C; source temperature 150 °C. Data processing for deconvolution was performed with the MaxEnt 1^TM^ function on the MassLynx software (Waters Corporation, Milford, CT, USA) using the following parameters: Mass range 40,000–80,000 Da; damage model based on uniform Gaussian 0.85 Da width at half height; minimum intensity ratios for adjacent peaks, left 33% and right 33%; iterated to convergence. Mercaptoalbumin (HSA-SH) and thiolated albumin (+120 ± 2 Da, Thio-HSA) were detected and their intensities were used to calculate the relative abundances as previously described [21]. For all the tested samples, a coefficient of variation of 7.0 ± 1.1% (mean ± SEM) was calculated for the percentages of thio-HSA.

### 2.3. Identification of Thiol Compounds Bound to HSA 

Human plasma samples were initially purified using the Thermo Scientific Pierce™ Albumin Depletion Kit (Pierce Biotechnology, Rockford, IL, USA) to separate albumin protein from samples, following the manufacturer’s instructions. Briefly, after the equilibration of the resin, 50 µL of plasma samples diluted 2-fold in binding/wash buffer were incubated for 2 min at room temperature. After centrifugation at 12,000× *g* for 1 min, the flow-through was applied again to the spin column, incubated for a maximal albumin binding, and centrifuged. The resin was washed four times to release unbound proteins by adding binding/wash buffer. To elute bound albumin, the resin was washed twice with 1.5 mol/L NaCl. The albumin fractions (400 µL) were then purified and concentrated using Amicon^®^ Ultra-0.5 centrifugal filter devices (30 KDa cutoff) according to the manufacturer’s instructions; centrifuged at 14,000× *g* for 2 min at room temperature, leaving approximately 100 µL of the sample above the filter; and the retained sample was diluted with 25 mmol/L phosphate buffer (PB) pH 8, for a total of three times. Finally, to recover the concentrated albumin, the filtering device was placed upside down in a clean microcentrifuge tube and centrifuged at 1000× *g* for 2 min. For each sample, the albumin concentration was determined, and all the samples were normalized to the same concentration (10 mg/mL) with 25 mmol/L PB pH 8. Thus, 100 µL of protein samples were treated with 2.5 mmol/L tris(2-carboxyethyl)phosphine (TCEP) for 30 min at 40 °C followed by a purification with filter devices centrifuged at 14,000× *g* for 35 min at room temperature. The ultrafiltrates under 30 kDa were vacuum dried, resuspended in 25 mmol/L PB pH 8, and derivatized with 0.5 mmol/L 7-chloro-N-[2-(dimethylamino)ethyl]-2,1,3-benzoxadiazole-4-sulfonamide (DAABD-Cl) for 20 min at 50 °C. The reaction was stopped with a final concentration of 0.05% FA before the MS analysis. A standard curve was prepared by serial dilution into 25 mmol/L PB pH 8 of cysteine or homocysteine treated with DAABD-Cl. 

MS analysis was performed, according to the protocol described by Nakashima et al. [23], with slight modifications, using the Xevo TQ-S micro triple quadrupole mass spectrometer coupled with the ACQUITY UPLC^®^ M-Class system (Waters Corporation, Milford, CT, USA). Albumin samples were diluted 20-fold in H_2_O/FA 0.1% and 2 µL were injected and separated on the iKey Peptide BEH C18 column, 130 Å 1.7 µm, 150 µm × 100 mm (Waters Corporation, Milford, CT, USA) at the flow rate of 3 µL/min and 40 °C. A gradient of solvent A (H_2_O/FA 0.1%) and solvent B (ACN/FA 0.1%) was applied with a total run time of 20 min as follows: 0–2.5 min at 3% B; 2.5–7.5 min linear increase from 3 to 97% B; 7.5–8.5 min 97% B; 8.5–9.5 min 3% B. A trapping configuration was set before the analytical separation using the ACQUITY UPLC M-Class Symmetry C18 Trap Column, 100 Å, 5 µm, 300 µm × 50 mm (Waters Corporation, Milford, CT, USA) with the isocratic trapping flow rate at 30 µL/min 99.5% A/0.5% B and the trapping time of 1 min. LC-MS/MS analysis was performed in multiple reaction monitoring (MRM) mode, in positive ion mode (cone voltage 30 V; collision energy for DAABD-cysteine 35 eV; collision energy for DAABD-homocysteine 32 eV). The parent ion scan was 72.2 *m*/*z*. The MRM mode scan was as follows: DAABD-cysteine 390.2- > 72.2 *m*/*z*; DAABD-homocysteine 404.2- > 72.2 *m*/*z*. Each transition had a dwell time of 0.165 s. The analytical software TargetLynx (Waters Corporation, Milford, CT, USA) was used for data processing. 

A standard curve was prepared by serial dilution of DAABD-cysteine to obtain the final concentrations of 4.2, 42, 420, and 840 fmol/µL. Linear regression of all calibration curves was performed using a 1/× weighting (origin excluded). The calibration curve for DAABD-cysteine, analyzed in duplicate, resulted in an R2 of 0.998 with residual standard deviation (RSD) within ±20% for the LLOQ (4.2 fmol/µL) and ±15% of the nominal value for the other calibration standards tested. The reproducibility of the measurement was evaluated in a pool of samples analyzed 6 times, resulting in a coefficient of variation (CV) of 1.84%. Signal to noise (S/N) was >12,000 in all the tested samples. Similarly, a standard curve was prepared by serial dilution of DAABD-homocysteine to have the final concentrations of 52.5, 105, 210, 420, and 840 fmol/µL. The calibration curve for DAABD-cysteine resulted in an R2 of 0.999 with RSD within ±20% for the LLOQ (52.5 fmol/µL) and ±15% of the nominal value for the other calibration standards tested, 5.52% of CV for replicate analyses, and S/N > 500 for all tested samples. 

### 2.4. Antioxidant Activity Evaluation by Means of TRAP Assay

Antioxidant activity was evaluated by means of the fluorimetric TRAP assay monitoring the 2-electron oxidation of 2′,7′-dichlorofluorescin (DCFH), prompted by AAPH as the radical initiator, through the highly fluorescent compound 2′,7′-dichlorofluorescein (DCF) measurement, as previously described [24]. Briefly, the DCFH_2_ was prepared starting from 2’,7’-dichlorodihydrofluorescein diacetate (DCFH_2_-DA) by basic hydrolysis [24]; 40 µL of the plasma samples were spiked with DCFH_2_ and AAPH at the final concentration of 10 µmol/L and 10 mmol/L, respectively. Samples were loaded in triplicate on a 96-well plate (BRANDplates pureGrade, BRAND^®^, 97877 Wertheim, Germany) and the fluorescent compound DCF was monitored at 37 °C using a multilabel multitask plate reader (Victor-1420 Multilabel Counter; Wallac, Turku, Finland) setting the excitation and emission wavelengths at λex 485 nm λem at 535 nm, respectively. The antioxidant activity was finally evaluated by calculating the lag-phase (min) induced before the substrate oxidation.

### 2.5. Regeneration of Mercaptoalbumin

Regeneration of mercaptoalbumin was performed by using human serum albumin from Grifols (Milan, Italy) and measuring the relative content of the two isoforms of albumin by MS intact protein analysis as already described [21]. One milliliter of albumin (60 mg/mL) was treated with N-Acetyl-Cysteine (NAC) (100 µg/mL) as previously described [24]. The reaction mixtures were incubated for 60 min at 37 °C under stirring (85 rpm) in a thermomixer. Then, in order to remove NAC from the albumin fractions, the samples were purified and concentrated using Amicon^®^ Ultra-0.5 centrifugal filter devices (30 KDa cutoff) according to the manufacturer’s instructions. Briefly, 500 µL of sample were added to the ultra-filter device, which was centrifuged at 14,000× *g* for 20 min at room temperature, leaving approximately 50 µL of the sample above the filter. The remaining albumin fraction was brought up to 500 µL twice with the addition of sterile water and once with serum-free medium. Finally, the recovered concentrated albumin was brought up to 500 µL with the addition of serum-free medium, and the concentration was determined by the Bradford method [25].

### 2.6. Cell Culture and Treatment

The HL-1 cardiomyocytes, a kind gift of Prof. W.C. Claycomb, (LSU Health Sciences Center, New Orleans, LA, USA), were cultured in complete Claycomb medium supplemented with 10% FBS (Sigma-Aldrich, Milan, Italy), 2 mmol/L L-glutamine (Thermo Fisher Scientific, Milan, Italy), and 100 µmol/L norepinephrine (Sigma-Aldrich, Milan, Italy) according to Prof. Claycomb’s instructions [26]. HL-1 cells (4 × 10^4^) were seeded onto a 24-well plate and were grown for 48 h in complete medium. Cells were pretreated with serum-free medium containing 2% w/v HSA (native or NAC-treated) for 16 h, followed by treatment with H_2_O_2_ 200–800 µmol/L or vehicle (control cells) for 7 h. 

### 2.7. MTT Assay

The methylthiazolyldiphenyl-tetrazolium bromide (MTT) assay is based on the protocol first described by Mosmann [27]. Briefly, cells were incubated for 30 min at 37 °C with 0.1 mg/mL of MTT (Sigma-Aldrich, Milan, Italy), dissolved in serum- and phenol-free medium. At the end of the incubation, cells were dissolved in DMSO. Absorbance was recorded at 550 nm using the microplate spectrophotometer system (Mithras LB940, Berthold Technologies, Bad Wildbad, Germany). Data are expressed as absorbance values (ratio vs. control cells).

### 2.8. Statistical Analysis

Clinical data from healthy subjects (control) and HF patients were analyzed using SAS v9.4 (SAS Institute, Cary, NC, USA) to subdivide HF patients according to NYHA class (III vs. IV). Univariate analysis was performed by ANOVA to identify statistically different variables among groups while Pearson correlation was used to identify a possible correlation between thiolated HSA (thio-HSA) and clinical variables. The general linear model (GLM) was used to highlight the trend of an increase of thio-HSA with the severity of the disease, both as univariate analysis and as multivariate analysis taking into consideration differences of age, hypertension, and dyslipidemia due to their significant correlation with thio-HSA. 

Regarding the set of data related to small thiols bound to HSA and TRAP assay, univariate analysis by unpaired two-way Student’s t test was performed to compare control subjects and HF patients. Pearson correlation analysis was performed to test the relationship between low-molecular-weight thiols bound to HSA and TRAP or thio-HSA.

In vitro data were analyzed with GraphPad Prism v5.03 using two-way ANOVA to test the interaction between H_2_O_2_ and thio-HSA or NAC-HSA treatment. 

Normality assessment was carried out by Kolmogorov–Smirnov tests. Data are expressed as mean ± standard deviation (SD), and *p* values of <0.05 were considered significant.

## 3. Results

### 3.1. Analysis of Thiolated Albumin in Heart Failure (HF) Patients

**Figure 1:**
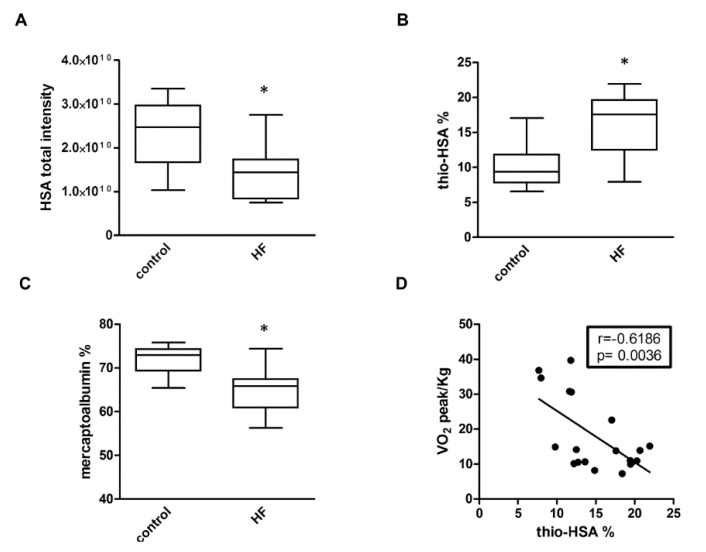
Thiolated albumin in heart failure (HF) patients. Total HSA (**A**), percentages of thiolated albumin (**B**), and mercaptoalbumin (**C**) in plasma of controls and HF patients. * *p* < 0.05 vs. control. (**D**) Correlation between thio-HSA and peak VO_2_.

The fraction of thiolated albumin (thio-HSA), with respect to all the albumin isoforms, was measured with the described MS-based method (Figure S1) in human plasma samples from a cohort of HF patients and controls (Table S1). First of all, we observed a global decrease of albumin content in the plasma of HF patients in respect to control subjects (1.44 × 10^10^ ± 0.6 × 10^10^ and 2.37 × 10^10^ ± 0.74 × 10^10^ respectively, *p* = 0.0019). In addition, comparing the percentages of thio-HSA, we observed an increase of thio-HSA in HF patients (16.07 ± 4.381%, and 10.21 ± 3.023%, respectively, *p* = 0.0013) and, consequently, a decrease in the reduced form of albumin, mercaptoalbumin (71.83 ± 3.214%, 64.62 ± 5.030%, in healthy subjects and HF patients, respectively, *p* = 0.0006) (Figure 1).

Moreover, subdividing HF patients according to the NYHA classification, we evidenced a higher percentage of thio-HSA in patients with the higher degree of severity (14.8 ± 5.231%, 17.18 ± 3.452%, NYHA class III and class IV, respectively). Univariate statistical analysis revealed both a between-group difference (ANOVA, *p* = 0.001) and a trend of increase with severity (GLM, *p* = 0.0003). In order to identify confounding factors, we performed Pearson correlation analysis of thio-HSA with clinical and demographic parameters. Considering that thio-HSA correlates with age (r = 0.738, *p* < 0.0001), the presence of hypertension (r = 0.566, *p* = 0.0026), and dyslipidemia (r = 0.447, *p* = 0.0219), we performed a multivariate analysis considering these confounding factors, and we confirmed the results of the univariate analysis (ANOVA, *p* = 0.05; GLM, *p* = 0.0138). Of note, correlation with age was present also in control subjects (r = 0.6367, *p* = 0.0478), thus suggesting a link between aging and oxidative stress-related processes.

We then assessed the relationship between thio-HSA and cardiopulmonary functional parameters. Interestingly, a strong inverse correlation was also observed between thio-HSA and peak VO_2_/kg (Figure 1D), an index of oxygen consumption that is considered to be associated with HF severity [28].

### 3.2. Identification of Low-Molecular-Weight Thiols Bound to Albumin

**Figure 2:**
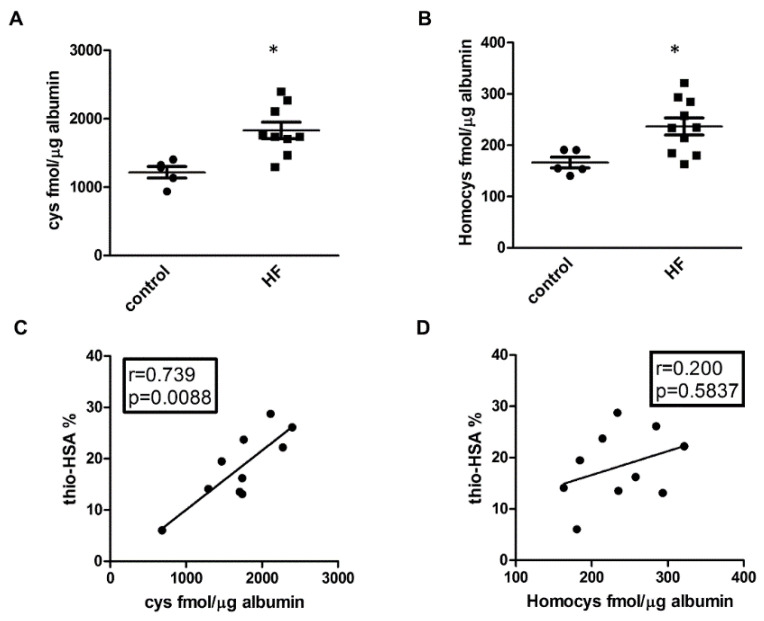
Identification of low-molecular-weight thiols bound to albumin. (**A**,**B**) Quantification of cysteines bound to HSA (cys) and homocysteines bound to HSA (Homocys). * *p* < 0.05 vs. control. (**C**,**D**) Correlation between S-thiolated albumin (thio-HSA%) and cysteines or homocysteines bound to HSA.

In order to further characterize the thiolated albumin in the plasma of HF patients, we took advantage of a MS-based method, described in the materials and methods section, that allowed us to discriminate between cysteine and homocysteine bound to HSA. Thus, we measured the content of cysteine and homocysteine bound to HSA in a group of HF patients with low levels of VO_2_/kg (<12, *n* = 10), index of severe HF, and a control group of sex- and age-matched healthy subjects (*n* = 5), as described in Table S2. This analysis revealed that both cysteine and homocysteine bound to HSA are increased in HF patients with respect to healthy subjects (Figure 2A,B and Figure S2), but the levels of cysteine were much higher than that of homocysteine. Indeed, cysteine represents 87.92% and 87.34% of the low-molecular-weight thiols bound to HSA in healthy and HF, respectively (*p* = NS). Moreover, we tested the correlation of the levels of low-molecular-weight thiols with the thio-HSA fraction. A strong correlation with thio-HSA was demonstrated only for cysteine and not homocysteine (Figure 2C,D), further confirming the contribution of cysteine in thiolation of albumin in HF.

**Figure 3:**
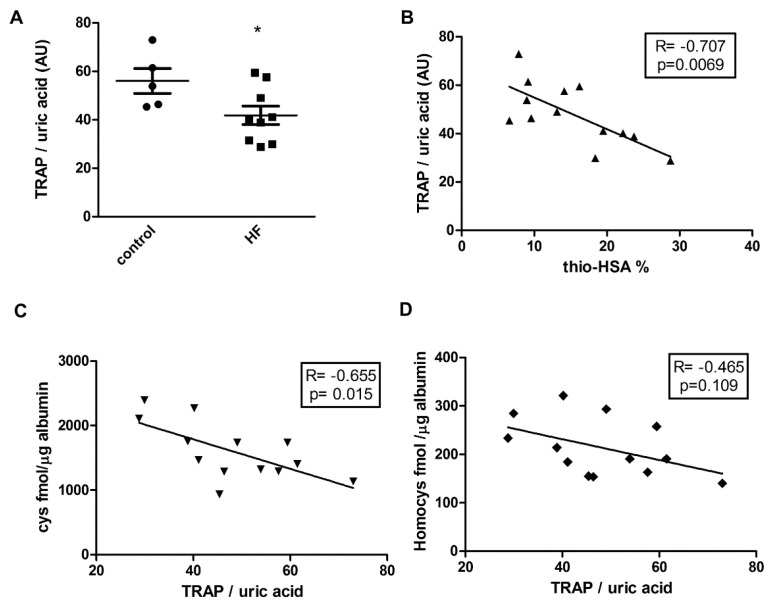
Correlation between S-thiolation and antioxidant properties of HSA. (**A**) Results from the TRAP assay performed on control and HF subjects. Data are expressed as lag-phase of TRAP assay/uric acid plasma concentration (**B**–**D**) Correlation between antioxidant activity and percentage of thiolated albumin, cysteine, and homocysteine bound to HSA. * *p* < 0.05 vs. control.

An evaluation of antioxidant activity performed by means of the TRAP assay normalized on the basis of the uric acid content showed a significant difference between control and HF subjects in terms of the lag-phase (min) induced before the substrate oxidation (Figure 3A and Figure S3). Further, in order to evaluate the relationship between the antioxidant activity of HSA and thiolation of albumin, we performed correlation analysis of the results from the TRAP assay and HSA thiolation. Of note, a strong correlation between antioxidant activity and percentage of thiolated albumin (Figure 3B) was observed, but a significant correlation with antioxidant activity was evidenced only for cysteines bound to HSA and not homocysteines (Figure 3C,D).

### 3.3. Thiolated Albumin Loses Its Protective Effects Against H_2_O_2_ in Cardiomyocytes

**Figure 4:**
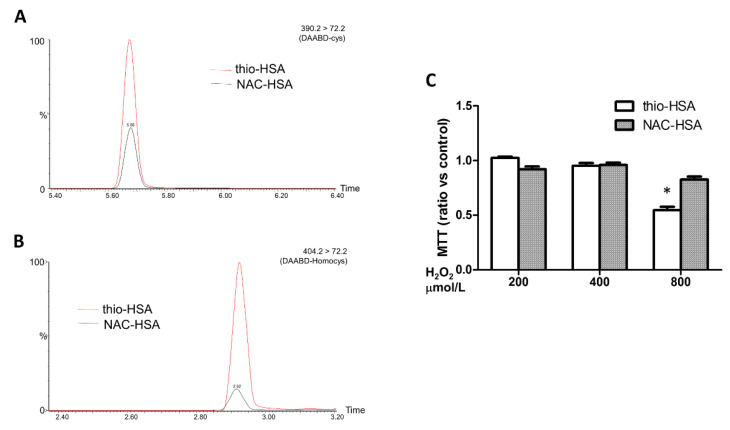
Biological effects of S-thiolated albumin. (**A**,**B**) Effects of NAC treatment on the levels of low-molecular-weight thiols bound to HSA. Representative chromatograms of the analysis of low-molecular-weight thiols bound to HSA after derivatization with DAABD-Cl are shown in panel A for cysteine bound to HSA (DAABD-cys) and in panel B for homocysteine bound to HSA (DAABD-homocys). (**C**) Cell viability of HL-1 cells pretreated with thio-HSA or NAC-HSA and subsequently treated with H_2_O_2_ at different concentrations for 7 h. * *p* > 0.05 by two-way ANOVA.

We further examined the effect of thiolation on the functional properties of HSA. To address this issue, we used a purified HSA with a high percentage of thio-HSA (control HSA) and we compared its effects with those of the same purified HSA treated with NAC (100 µg/mL, NAC-HSA) to regenerate mercaptoalbumin. We confirmed that the treatment with NAC reduces the content of thio-HSA (from 37 ± 1.86% to 14.03 ± 1.24%), and we further demonstrated that both cysteine and homocysteine bound to HSA were reduced (Figure 4A,B). 

We then compared the protective antioxidant properties of control HSA and NAC-HSA in HL-1 cardiomyocytes treated with H_2_O_2_. After 7 h of treatment with H_2_O_2,_ cells pretreated with control HSA showed a higher decrease of cell viability in respect to cells pretreated with NAC-HSA (Figure 4C), suggesting that reversion of thio-HSA to mercaptoalbumin restores the antioxidant properties of HSA. 

## 4. Discussion

In the present study, we found for the first time that S-thiolation of albumin is increased in the plasma of HF patients. Both cysteine and homocysteine were identified as the low-molecular-weight thiols bound to HSA, with the levels of S-cysteine being higher than that of homocysteine. Further, the increase in S-thiolated albumin correlates with an impairment of the plasma antioxidant activity, thus suggesting that S-thiolation induced changes in the structure and function of HSA [29], which likely contribute to the progression of the disease. Interestingly, a significant inverse correlation was found between thio-HSA and peak VO_2_, which is considered the most objective functional method to assess the cardiopulmonary exercise capacity due to its ability to be measured reproducibly and to accurately reflect HF severity, as increasingly recognized and endorsed by scientific statements. Of note, peak VO_2_ is also an important predictor of prognosis in HF patients and recent studies have demonstrated that peak VO_2_ potently stratifies the risk of contemporary HF (reduced and preserved ejection fraction HF) populations [30]. We therefore might hypothesize that thio-HSA might represent a potential circulating marker of HF diagnosis and prognosis.

Biological thiols, cysteine and homocysteine, play a key role in maintaining an internal redox homeostasis. These thiols, together with cysteinyl glycine, glutathione (GSH), and γ-glutamyl cysteine, belong to a class of highly reactive compounds largely involved in the maintenance of redox homeostasis, whose identity, levels, and variety depend on the biome. In our body, the intracellular thiol concentration reaches millimolar. In contrast, in the extracellular environment, especially in plasma, the thiol compounds are much lower [31]. Specifically, in human blood, the concentration of total plasma cysteine is 200–300 µmol/L, whereas that of homocysteine is around 10 µmol/L [29], thus explaining the prevalence of cysteinylation over homocysteinylation on albumin. 

We also provided the evidence that S-thiolated albumin did not prevent the decreased viability of the cardiomyocytes HL-1 induced by the oxidant hydrogen peroxide, whilst mercaptoalbumin did. Oxidized albumin is known to also mediate inflammatory reactions and cellular senescence in endothelial cells [32,33,34] and also in cardiomyocytes once modified by glycation [22]. Here, we showed that the conversion of S-thiolated albumin to mercaptoalbumin prevented the deleterious effect of hydrogen peroxide on cells, likely due to the capacity of Cys34 to scavenge the oxidant species. We indeed recently demonstrated that the restoration of Cys34 by NAC is able to selectively break the disulfide bond of thiolated albumin, leading to the disulfide NAC-Cys and the free form of Cys34, which in turn further acts as an antioxidant [24].

Our findings provide new insights into structural alteration of HSA under physiological and pathophysiological conditions. Indeed, we found a positive correlation between thio-HSA and age, thus suggesting a link between aging and oxidative stress-based processes. In the context of HF, the interest in albumin has been regarded only in terms of quantity and not quality. Hypoalbuminemia is common in HF patients and this condition is present in HF patients with depressed systolic function [17] as well as with preserved systolic function [14,35,36]. Moreover, hypoalbuminemia can occur in stable chronic HF patients and acute HF patients, where it appears to be reliable in predicting mortality [16,17,36]. Accordingly, our results confirm that patients with HF have significantly lower levels of albumin with respect to control subjects, but, of novelty, the residual albumin is also heavily oxidized. Further, in the Cardiovascular Health Study, enrolling 5450 healthy individuals aged ≥65 years, hypoalbuminemia was found to be significantly associated with the risk of HF in 10 years [19]. Mechanisms leading to hypoalbuminemia have not been specifically studied in patients with HF; malnutrition and liver dysfunction may be a potential cause of hypoalbuminemia [35]. Interestingly, systemic inflammation may reduce the serum albumin concentration independently of malnutrition [37]. The activation of the inflammatory response leads indeed to relevant changes in plasma proteins, cytokines, and complements, and albumin is a negative-phase protein whose concentration decreases in response to inflammation [38]. In support of this, in clinical states associated with chronic inflammation and oxidative stress, hypoalbuminemia is prevalent [39]. 

HF is certainly characterized by both inflammation and oxidative stress, which are also involved in the development and progression of the disease [40]. In this regard, in the last decades, several studies have tried to target these factors, with the aim of improving the outcome in patients with HF, but with very limited success (reviewed in [40]). Although the findings of clinical trials aimed at reducing reactive oxygen species production and increasing exogenous antioxidants have been disappointing, targeting oxidative stress, specifically the endogenous antioxidant capacity, in HF still represents a valuable alternative. Particularly noteworthy has been the observation that increasing the antioxidant capacity, by bolstering endogenous GSH levels, results in improved patient outcomes [41,42]. Until now, NAD+ and GSH have been considered the major endogenous antioxidants in mammalian cells; both have been shown to be associated with HF in the experimental and clinical setting, and their supplementation has been found to improve cardiac function in experimental models of HF [43]. 

Thus, our study adds albumin to the list of potential markers of HF severity, and of the putative drug-targetable endogenous antioxidants. Indeed, albumin accounts from 40% to 70% of the total antioxidant capacity of plasma [24]. To this regard, our in vivo and in vitro findings, although suggestive of a role of Cys34 in the antioxidant activity, do not allow us to rule out additional antioxidant mechanisms, such as those related to the ligand-binding capacities of HSA. To the best of our knowledge, there are no validated and established methods to measure the antioxidant plasma capacity based on oxygen radicals. For this reason, most of the antioxidant tests are based on radical generators (mainly aza-initiators that decompose thermically time dependently to generate carbon radicals), such as that used in the TRAP assay, which is recognized as the most established method for measuring the plasma antioxidant capacity.

## 5. Conclusions

Our findings highlight a novel role for S-thiolated albumin in HF. It is well known that hypoalbuminemia in HF patients is an independent cardiovascular risk factor; however, a mechanistic explanation between hypoalbuminemia and vascular injury has not yet been provided. We hypothesized that oxidized modifications of albumin, i.e., thiolation, explain the role of hypoalbuminemia as a predictor of cardiovascular morbidity and mortality. Although further studies are required to confirm these observations in larger studies, our results may provide a new paradigm of the proinflammatory effect of *S*-thiolated HSA, which may be exploited in the prevention of and therapy for HF and other chronic inflammatory diseases.

## Supplementary Materials

The following are available online at https://www.mdpi.com/2076-3921/9/8/763/s1. Figure S1: Representative spectra of HSA isoforms in a control subject and a HF patient. Figure S2: Representative chromatogram of the measurement of cysteine (A) and homocysteine (B) bound to HSA in a control subject and a HF patient. Figure S3: Fluorescent kinetics of the TRAP assay performed on plasma from individual subject (A, control subjects; B, HF patients), before normalization. Table S1: Clinical characteristics of heart failure patients and control subjects for HSA thiolation analysis. Table S2: Clinical characteristic of patients and healthy subjects employed for the measurement of thiols bound to HSA and TRAP assay.

## Data Sharing

Data collected in the study will be made available using the data repository Zenodo (https://zenodo.org/) with restricted access upon request to direzione.scientifica@ccfm.it.
