# Supplementary material for: S-Thiolation Targets Albumin in Heart Failure
Source: Antioxidants (Basel). 2020 Aug 17;9(8):763. doi: 10.3390/antiox9080763 (PMC7463808; doi:10.3390/antiox9080763)
Supplement: Supplementary file 1 [file antioxidants-09-00763-s001.zip › supplementary materials/Table S1.docx]

**Table S1.** Clinical characteristics of heart failure patients and control subjects for HSA thiolation analysis.

|  | **Healthy subjects (n=10)** | **HF patients (n=15)** |
| --- | --- | --- |
| **Characteristics** |  |  |
| **Age** | 56.3±4.7 | 67.7±9.1 |
| **Gender (m/f)** | 7/3 | 11/4 |
| **Hypertension** | 0/10 | 11/15 |
| **Dyslipidemia** | 0/10 | 9/15 |
| **Smoke** | 1/10 | 5/15 |
| **DM II** | 0/10 | 0/15 |
| **BMI** | 25.66±3.42 | 26.64±3.98 |
| **FE (%)** | N/A | 33.55±10.78 |
| **BNP (pg/mL)** | N/A | 546.8±564.5 |
| **NYHA (III/IV)** | N/A | 7/8 |
| **DLCO %** | 94.21±21.06 | 68.6±15.2 |
| **VO2_peak_Kg (mL/min/Kg)** | 34.58±8.46 | 13.04±6.43 |

HF, Heart failure; NYHA, New York Heart association class; DM, diabetes mellitus; BMI, body mass index; BNP, brain natriuretic peptide; FE, ejection fraction;
