# Supplementary material for: S-Thiolation Targets Albumin in Heart Failure
Source: Antioxidants (Basel). 2020 Aug 17;9(8):763. doi: 10.3390/antiox9080763 (PMC7463808; doi:10.3390/antiox9080763)
Supplement: Supplementary file 1 [file antioxidants-09-00763-s001.zip › supplementary materials/Table S2.docx]

**Table S2**. Clinical characteristic of patients and healthy subjects employed for the measurement of thiols bound to HSA and TRAP assay.

|  | **Healthy subjects (n=5)** | **HF patients (n=10)** |
| --- | --- | --- |
| **Characteristics** |  |  |
| **Age** | 56±1.4 | 59.2±7.9 |
| **Gender (m/f)** | 5/0 | 10/0 |
| **Hypertension** | 0/5 | 4/10 |
| **Dyslipidemia** | 0/5 | 5/10 |
| **Smoke** | 0/5 | 3/10 |
| **DM II** | 0/5 | 4/10 |
| **BMI** | 24.51±2.29 | 29.17±7.11 |
| **FE (%)** | N/A | 26.43±6.86 |
| **BNP (pg/mL)** | N/A | 795±313 |
| **VO_2__peak_Kg (mL/min/Kg)** | N/A | 10.14±1.54 |

HF, Heart failure; NYHA, New York Heart association class; DM, diabetes mellitus; BMI, body mass index; BNP, brain natriuretic peptide; FE, ejection fraction;
