# Supplementary figures and images for: S-Thiolation Targets Albumin in Heart Failure
Source: Antioxidants (Basel). 2020 Aug 17;9(8):763. doi: 10.3390/antiox9080763 (PMC7463808; doi:10.3390/antiox9080763)

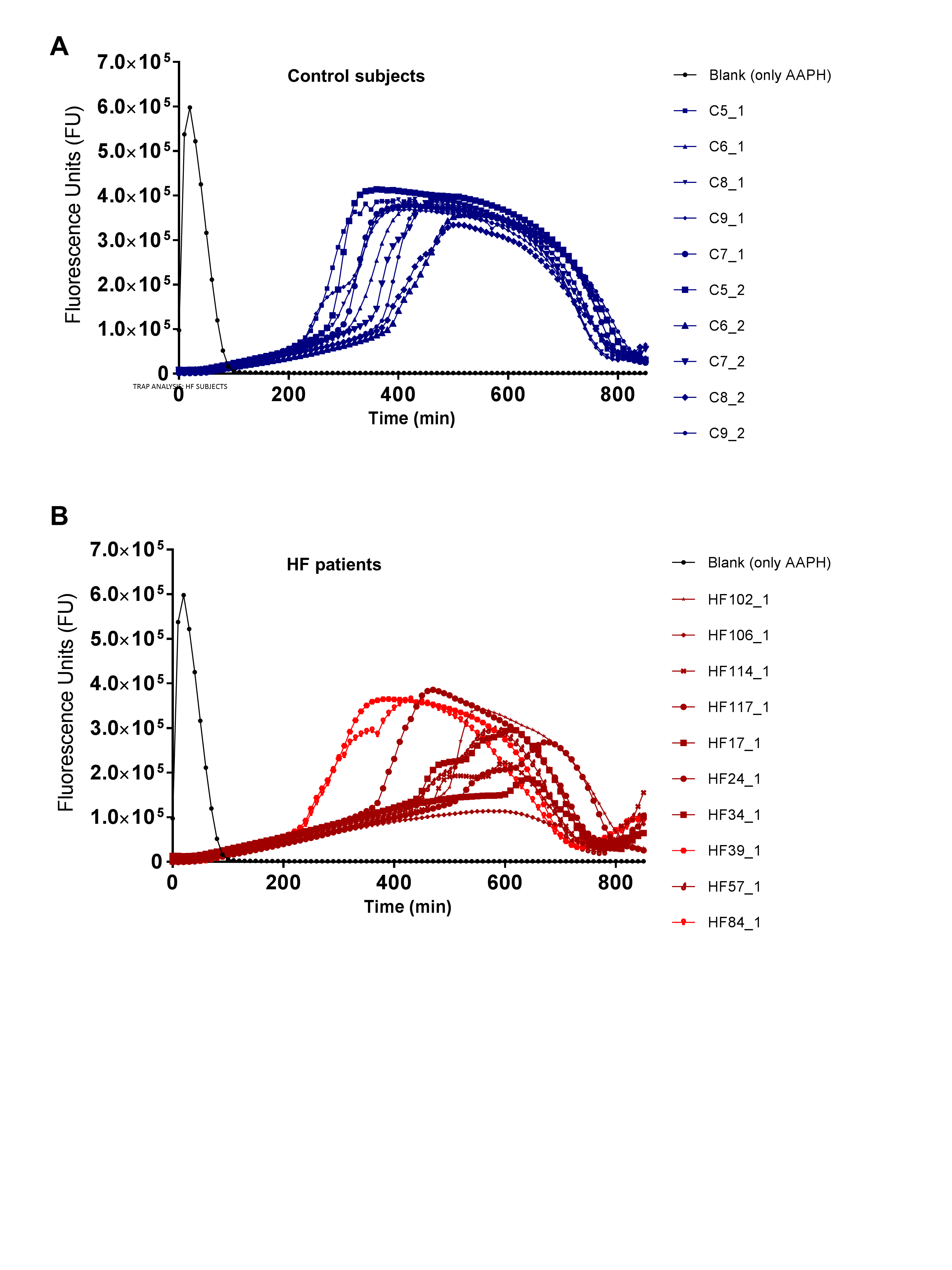

Supplement: Supplementary file 1 [file antioxidants-09-00763-s001.zip › supplementary materials/Figure S3.tif]
